# The Evaluation of Emotional Intelligence by the Analysis of Heart Rate Variability

**DOI:** 10.3390/s23052839

**Published:** 2023-03-05

**Authors:** Gangyoung Lee, Sung Park, Mincheol Whang

**Affiliations:** 1 Department of Emotion Engineering, Sangmyung University, Seoul 03016, Republic of Korea; 2 Department of Human-Centered Artificial Intelligence, Sangmyung University, Seoul 03016, Republic of Korea

**Keywords:** emotion intelligence, heart rate variability, physiological measures

## Abstract

Emotional intelligence (EI) is a critical social intelligence skill that refers to an individual’s ability to assess their own emotions and those of others. While EI has been shown to predict an individual’s productivity, personal success, and ability to maintain positive relationships, its assessment has primarily relied on subjective reports, which are vulnerable to response distortion and limit the validity of the assessment. To address this limitation, we propose a novel method for assessing EI based on physiological responses—specifically heart rate variability (HRV) and dynamics. We conducted four experiments to develop this method. First, we designed, analyzed, and selected photos to evaluate the ability to recognize emotions. Second, we produced and selected facial expression stimuli (i.e., avatars) that were standardized based on a two-dimensional model. Third, we obtained physiological response data (HRV and dynamics) from participants as they viewed the photos and avatars. Finally, we analyzed HRV measures to produce an evaluation criterion for assessing EI. Results showed that participants’ low and high EI could be discriminated based on the number of HRV indices that were statistically different between the two groups. Specifically, 14 HRV indices, including HF (high-frequency power), lnHF (the natural logarithm of HF), and RSA (respiratory sinus arrhythmia), were significant markers for discerning between low and high EI groups. Our method has implications for improving the validity of EI assessment by providing objective and quantifiable measures that are less vulnerable to response distortion.

## 1. Introduction

The term ‘emotional intelligence’ (EI) has been popular with the general public since Goleman [1] wrote the book *Emotional Intelligence: Why it can Matter More than IQ* in 1995 [2]. Salovey and Mayer [3] first defined EI as “the ability to monitor one’s own and others’ feelings and emotions, to discriminate among them and to use this information to guide one’s thinking and actions.” By definition, EI includes the elements of intrapersonal (own feelings) and interpersonal (others’ feelings) skills [4] related to social intelligence [5]. EI has also been conceptualized as an intertwined relationship between affect and cognition [2]. For example, EI dimensions are strongly associated with academic success [6]. Decades of research in psychology have concluded that EI is critical for productivity and personal success [7].

### 1.1. Emotional Intelligence Models

Intelligence researchers have proposed and verified EI models with varying emphasis and purposes. Table 1 outlines the prominent EI models compared to our proposed model. The Mayer–Salovey–Caruso Emotional Intelligence Test (MSCEIT) [8] consists of emotion-based problem-solving items designed based on the four-branch model. The four-branch model outlines the ability to recognize, integrate, and understand emotions, which consists of perceiving, using, understanding, and managing them. The MSCEIT is designed based on the premise that EI is a type of intelligence. The MSCEIT is considered an ability test that produces a score for four branches of emotional intelligence, in addition to a total score. However, the accuracy of the MSCEIT has been criticized due to a lack of normalization of the emotional stimulus. Although certified emotion experts manually assessed the scores, the scores could not ensure objectivity.

The Emotional Competency Inventory (ECI) was created by Goleman in 1999 [9], followed by the Emotional and Social Competency Inventory (ESCI) in 2007 [10], an updated version of the ECI. Such measurement tools aim to provide behavioral measures through a 360-degree survey designed to evaluate 12 social and emotional competencies. The EI competency model comprises self-awareness, self-management, social awareness, and relationship management. Although the inventories of Goleman, based on mixed models, have the clear purpose of assessing a person’s leadership, the survey is vulnerable to response distortion. Moreover, the inventories did not focus on emotion recognition abilities.

**Table 1:** The strengths and weaknesses of each EI test.

EI Test	Strengths	Weaknesses
MSCEIT [8]	Considers the emotional problem-solving ability and includes visual stimuli (photos and facial expressions)	Estimates score intervals that are not standardized and the stimuli presented have no intensity levels
ECI [9]	Measures leadership performance by evaluating abilities and skills based on EI	As it is evaluated by a written questionnaire, it is vulnerable to response distortion
EQ-i [11]	Evaluation based on only self-awareness as a characteristic model	The emotional level and resolution in the measurements are low and vulnerable to response distortion
Analysis of HRV and dynamics (**our model**)	Evaluates the EI level based on physiological measures. The stimuli and questionnaire are mapped on a two-dimensional emotion model. The stimuli are segmented according to emotional intensity	The level of emotional intelligence in each emotional domain needs further investigation

EQ-i [11] is a mixed model of EI developed by Reuven Bar-On that consists of 15 social and emotional competencies. Intrapersonal model factors such as emotional self-awareness, independence, and assertiveness are included. In EQ-i, the emotional level and resolution in the measurements were low and vulnerable to response distortion.

Overall, the EI tests have heavily relied on a written questionnaire, which is limited by the individual difference in the interpretation of the questions, resulting in response distortion. To overcome such weaknesses, physiological measurements such as HRV can provide precise, objective, and quantifiable data to verify an individual’s EI. Our proposed assessment model emphasizes the use of HRV and vagal tone measurements, which necessitate a comprehensive understanding of these physiological indicators.

### 1.2. Vagal Tone

Emotional regulation, one of Mayer’s four-branch models, is the critical ability to regulate one’s emotions reflexively and to promote emotional and intellectual improvement [12]. This involves balancing arousal and valence states to manage and cope with emotions.

Stephen Porges’ polyvagal theory [13] suggests that the parasympathetic nervous system (PSNS) of the autonomic nervous system (ANS) maintains a constant body environment by controlling the organs and hormones in the external environment. Two independent vagus pathways are present in the PSNS branch. First, only mammals and humans have the myelinated vagus nerve, which begins in the nucleus ambiguus (NA) and connects to the sinoatrial node (SA) of the cardiac. The vagus nerve is responsible for social engagement.

Second, most animals have the unmyelinated vagus nerve, which begins in the dorsal motor nucleus (DMNX). If we perceive danger, the sympathetic nervous system (SNS) is activated, and metabolism increases to cope with external threats. Vagal tone is known to correlate positively with emotional regulation [14]. Since SNS also has an antagonistic relationship with PSNS, it is critical to investigate cardiac (i.e., HRV) indexes to assess the ability of a person to regulate emotion. Because regulating emotion is a crucial component of EI, we analyzed physiological measures, such as HRV and dynamics, in our study, as described in the next section.

### 1.3. Emotion and Dynamics of Heart Rate Variability

HRV analysis provided critical insights into emotion research and recognition [15]. Non-invasive methods in wearable devices have been introduced, significantly improving the applicability of HRV-based emotion analysis [16,17]. However, emotional data, without considering an individual’s recognition and regulation capability, may fail to achieve validity and reliability. To address this concern, further investigation into HRV variables is necessary to capture individual differences in emotional regulation and recognition.

Dynamics is an indicator that can identify changes and fluctuations in the emotional state over a longer time scale [18]. Emotional response and self-regulation can be confirmed by the temporal dynamics of emotion. Our study adopted and measured the three factors of dynamics (variability, instability, and inertia) to overcome the limits of previous EI tests.

First, variability is an index that indicates the range and amplitude of emotional states over time. The higher the number and the more extreme the situation, the greater the emotional deviation and the lower the ability to self-regulate. It can be calculated as the degree of variance of the HRV. Second, instability is implied by the degree of emotional change between the present and the next moment. Higher numbers indicate greater emotional changes, meaning greater emotional anxiety and a lower ability to self-regulate. It can be calculated as the mean of the squared successive differences (MSSD) of HRV. Third, inertia is an index that indicates the residual degree of emotion, which predicts the intensity of the current emotional state from the emotional state of the previous moment. A high value indicates that the recovery rate of emotional homeostasis is relatively low. This can be calculated as the autocorrelation of the HRV.

### 1.4. Proposed Study

Thus far, we have compared emotional intelligence models that have attempted to assess the emotion recognition capability of participants. All models have a common weakness: they rely heavily on subjective evaluations of the participants. We propose a novel method to evaluate EI based on the physiological responses (HRV and dynamics) of the participants. To the best of our knowledge, this is the first study to analyze HRV to evaluate EI. To this end, we designed an emotion-eliciting stimulus based on a dimensional model. We improved the stimulus granularity by facial intensity. 

Four experiments were conducted to build on one another. The first two experiments (see Table 2) were designed to produce stimuli that would be used in the latter two experiments (see Table 3). We adopted the four emotional perception levels framework [19], which consists of “self-awareness (Level 1, 3),” “others-awareness (Level 2, 3),” and “distinguish expressed emotion (Level 3, 4)”, and used them as a theoretical framework in designing the experiment. 

In the first experiment, based on the IAPS model [20], we verified and selected photo stimuli to comprehensively evaluate the ability to recognize emotions (level 1) and express them well (level 3). In the second experiment, we chose stimuli that could comprehensively assess the ability to recognize the emotions of others (level 2) and express their own emotions well (level 3). Specifically, facial expressions were standardized based on the two-dimensional emotional model using avatars, and facial expression stimuli were defined by normalizing the intensity of the facial expression.

In the third experiment, using the stimuli produced by the previous experiments (#1 and #2), HRV and dynamic data reflecting emotion regulation were obtained. In the fourth experiment, the EI level was evaluated through self-reports using photos and avatars. We collected ECG data while the participants watched the emotion-eliciting video and identified the critical HRV and dynamics to classify EI based on this analysis.

## 2. Experiment #1: Selecting Photo Stimuli for Evaluation of Self-Aware Emotions

Among the four levels of emotional recognition, it was necessary to select stimuli to comprehensively evaluate the ability to recognize one’s emotions (Level 1) and express them well (Level 3). As stimuli for eliciting one’s own emotions, there are representative emotional photos, such as the International Affective Picture System (IAPS), but the accuracy of the participants’ recognition of emotions elicited by the photos is relatively low, with an average of 51% and the highest accuracy of 81% [21]. The IAPS also has low validity because it does not limit the exposure time of stimuli. We limited the exposure time to 300 msec and selected stimuli with a mean accuracy of 81% or above, which was the highest accuracy achieved by the IAPS.

### 2.1. Participants

Thirty-three participants were recruited to verify, standardize, and normalize the candidate stimuli. Ages of the participants ranged from 20 to 30 years (mean = 25.9, STD = 2.7), with 14 (42%) men and 19 (58%) women. All participants were briefed on the purpose and procedure of the experiment, and signed consent was obtained from them. The participants were then compensated for their participation by paying a fee.

### 2.2. Stimuli

We collected photos by referring to the stimuli model of the IAPS to evaluate self-awareness [20]. IAPS provides a set of emotional stimuli involving attention and emotion, ranging from scenes of nature to household objects. Because we aimed to select stimuli for the self-awareness of participants’ emotions, we excluded stimuli with facial expressions, thereby distinguishing from recognizing others’ emotions. A total of 120 candidate stimuli were selected, with 30 stimuli for each of the four emotional domains. The participants viewed the black cross at the center of the screen for 500 msec to fixate their gaze. They then viewed the candidate stimuli for 300 msec. We referred to the exposure time based on a previous study [22].

### 2.3. Experimental Protocol

**Figure 1:**
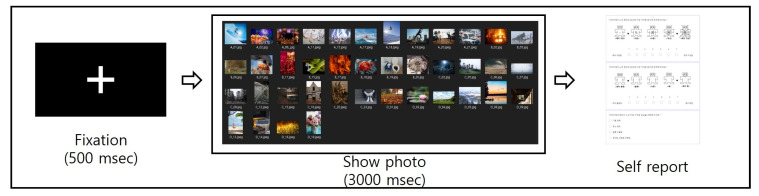
Experimental protocol for selecting stimuli for self-awareness.

Figure 1 outlines the experimental process and environment used in this study. The participants were seated one meter away from a 27-inch LCD monitor. The participants responded to a questionnaire after viewing each photo stimulus. They responded to 7-point Likert scales representing valence and arousal levels. A self-assessment manikin (SAM) [23] was provided as a guide. The participants then selected an emotion keyword representing each domain in the dimensional emotion (i.e., happiness and joy for HAHV (high arousal and high valence), anger and disgust for HALV (high arousal and low valence), sadness and depression for LALV (low arousal and low valence), and comfortable and stable for LAHV (low arousal and high valence)) in a multiple-choice format. Each participant viewed all 120 stimuli and responded to a questionnaire. By doing so, we were able to assess the ability of the participants to recognize their own emotions (Level 1) and express their emotions through a questionnaire (Level 3).

### 2.4. Result

Candidate emotional photos without facial expressions were exposed for a limited time (300 msec), and emotional photos higher than the target accuracy of 81% were selected, maintaining a rigid standard. In total, we selected 40 photos (10 per emotional domain). Table 4 outlines the average recognition accuracy of the ten photos in each emotional domain.

## 3. Experiment #2: Selecting Avatar Stimuli for Evaluation of Awareness of Others’ Emotions

It is necessary to choose stimuli that can comprehensively assess the ability to recognize the emotions of others (Level 2) and express their own emotions well (Level 3). We designed and calibrated avatar stimuli that mimicked human facial expressions. Because of the individual differences in the facial expressions of emotions, it is paramount to establish standardized criteria applicable to avatar stimuli. Specifically, we found the appropriate expression intensity for an avatar that the participant could recognize the most.

### 3.1. Participants

The participants were the same as in the first experiment.

### 3.2. Stimuli

**Figure 2:**
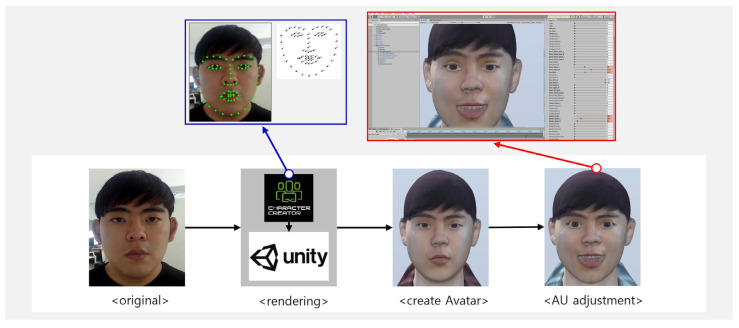
The process of creating a standardized facial expression avatar.

The facial expressions of each domain were made into avatars. We referred to the two-dimensional model of facial emotion [24], in addition to the action unit (AU) of the facial action coding system (FACS) [25,26,27]. Figure 2 outlines the process of creating a standardized, expressive facial avatar. The avatar was ported to Unity to create a face in Character Creator 3 by adjusting the AUs. Facial expression intensity was produced using the AU parameter provided by Unity at ten intensity levels, at equal intervals from 10 to 100.

### 3.3. Experimental Protocol

**Figure 3:**
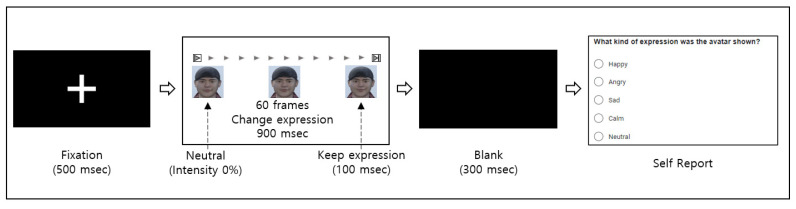
The experimental procedure for normalizing facial expression intensity.

Figure 3 outlines the experimental procedure for normalizing the intensity of the facial expression. The experimental environment was the same as that used in the first experiment. In random order, we displayed an avatar stimulus with varying emotion intensity. For 900 msec, the stimulus expression changed from neutral to target and was maintained with a 100 msec pause [28,29]. Before showing the avatar, a black cross was shown in the center of the screen for 500 msec, and the same black cross screen was shown for 300 msec after exposure to the stimulus.

Each participant viewed 40 stimuli, with ten intensity levels for each emotional dimension. After viewing, we first asked whether the participant could recognize the facial expressions of the avatar. We then asked which dimension the participant recognized. Participants chose four emotional (HAHV, HALV, LALV, and LAHV) and neutral domains. We assigned 10 points only when the participant recognized the facial expression and chose an emotional domain identical to the intended domain.

### 3.4. Result

**Figure 4:**
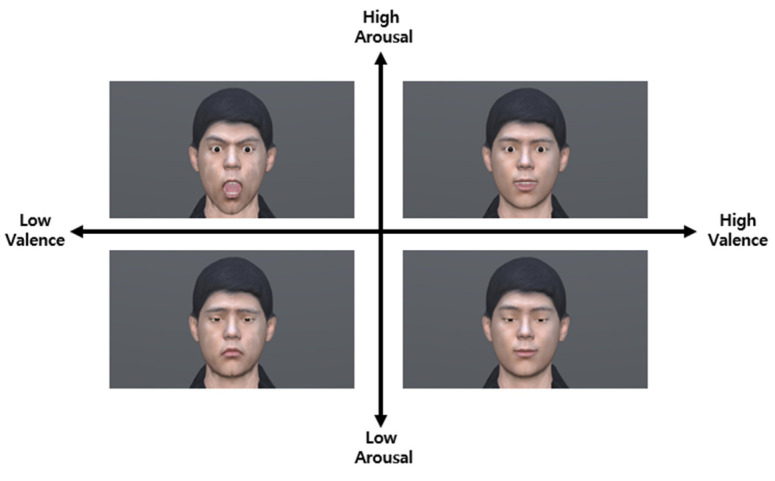
The standardized facial expression for each emotional domain.

Table 5 outlines the peak accuracy and the corresponding intensity of each emotional domain. As the emotional intensity increased in all emotional domains, the recognition accuracy as a target emotion increased linearly and gradually, and when a specific emotional intensity was reached for each emotional domain, the accuracy decreased linearly and gradually. To normalize the recognition accuracy of each emotional domain, the AU was adjusted to 10 intensities from minimal emotional expression (i.e., intensity 10%) to target emotional expression (i.e., intensity 100%). Normalization was performed based on an intensity of 80%, which is the intensity level recognized by most people. Representative facial expressions were recreated accordingly. Figure 4 shows the standardized facial expressions (intensity 100%) for each emotional domain.

## 4. Experiment #3: Obtaining Physiological Response (HRV and Dynamics)

It is paramount to create stimuli that elicit emotions to investigate EI by analyzing HRV and dynamics. In recent research, various types of content, such as photos [19], songs [30], and videos [31], have been used as stimuli to induce emotions. HRV requires the extraction of physiological signals for at least 3 min. To extract reliable and sufficient data, we designed video stimuli to last 6 min, two times the required duration (i.e., 3 min).

### 4.1. Participants

The participants were the same as in the first experiment.

### 4.2. Stimuli

**Figure 5:**
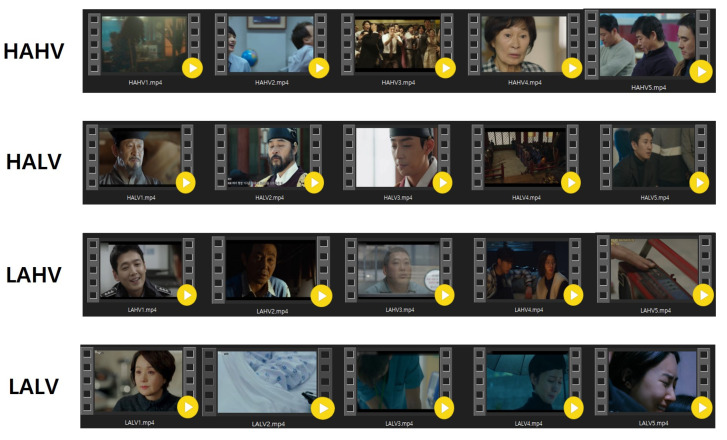
Five video clips per each emotional dimension.

Five video clips that would elicit emotions in the participants (e.g., dramas or films) were selected for each dimensional domain (see Figure 5). After watching each candidate video clip, participants were asked to rate their valence and arousal level using a 7-point Likert scale questionnaire. Additionally, they were asked to select a keyword representing one of the four dimensions. Only responses that matched the intended emotion were scored. We selected five video clips with the highest scores per dimension, where facial expressions corresponding to the target emotion were observed in over 50% of the frames. 

**Figure 6:**
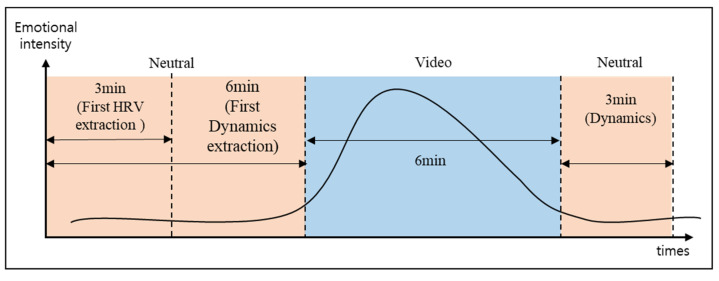
The emotional stimuli structure to extract HRV and dynamics.

Figure 6 outlines the emotional stimulus structure created to extract HRV and dynamics. To extract HRV, the length of the video clip was 6 min. In addition, to extract the dynamics, neutral video clips were attached 6 min before and 3 min after the stimuli. A neutral video was produced as an atypical black-and-white video to prevent drowsiness. In summary, we selected 20 stimuli, 5 per emotional domain and candidate emotion-eliciting stimulus. 

### 4.3. Experimental Protocol

The environment and questionnaire were the same as in the first experiment. To extract HRV and dynamics, electrocardiograms (ECG) were collected while participants watched the stimuli. ECG was collected at 500 Hz by attaching a sensor according to the standard limb guidance method (Lead I), amplifying it through ECG100C (BIOPAC Systems Inc., Goleta, CA, USA), and digitizing it with NI-DAQ-Pad9205 (National Instrument Inc., Austin, TX, USA). The collected ECGs were signal-processed using the LabVIEW 2010 software (National Instrument Inc., USA). For each candidate emotional stimulus, scores were assigned differentially according to the expected HRV value for the target emotion. The highest score was assigned to the stimulus that appeared the most. The score was given only when the arousal, valence, and emotional domains selected in the self-report matched the target emotions.

Finally, the stimuli with the highest scores were selected by adding the self-reported and HRV scores for each emotional domain. Scores were given by ranking the expected HRV that fit the target emotion of the produced stimuli.

### 4.4. Heart Rate Variability Analysis

Table 6 outlines the HRV variables used in this study. The ECG data were measured while the stimuli were presented for 15 min. HRV has two measurement methods: time-domain and frequency-domain methods. The time-domain indices quantify the amount of time-series data observed during the monitoring period. Frequency-domain indices are acquired by extracting the frequency band using the Fast Fourier transform (FFT) from time-series data observed during the monitoring period. This enabled the measurement of the ANS responses of participants exposed to stimuli. To measure the change in the serial heart rate, a 180 sec sliding window was used.

Humans have different HRVs according to gender, age, and environment. As a result, the normalization of HRV is required to reduce physiological differences and compare the differences between exposure to baseline and stimuli exposure. We normalized HRV based on changes in stimuli relative to the baseline. We acquired the baseline when the participants viewed a 6-min neutral video. The three dynamic factors (variability, instability, and inertia) were calculated for HRV. 

## 5. Experiment #4: EI Level Separation

The evaluation of EI thus far is vulnerable to response distortion because the main method is to segment responses using the self-reporting method. In addition, the granularity of the stimuli was not sufficient to accurately assess the EI of a person. Furthermore, the criteria for categorizing the evaluated EI levels were not grounded. In this study, we evaluated EI with subdivided emotional stimuli, and, using HRV, an involuntary indicator, we produced a robust criterion for classifying EI.

### 5.1. Participants

In this study, the power set of the two-tailed *t*-test (0.8 and α = 0.05, and d = 0.6) was analyzed using the a priori power analysis program G-power. The results suggest that N = 46 is required to obtain statistical power. As a result, 48 adults in their twenties (mean age = 27.4, STD = 2.7) were recruited, with 21 (44%) of them being men and 27 (56%) of them being women. To ensure the reliable perception of HRV and visual stimuli, participants with no medical history and a corrected visual acuity of 0.8 or greater without visual impairment were selected. We recommended that participants slept sufficiently and prohibited alcohol, caffeine, and smoking to ensure accurate self-report and ECG data. All participants were briefed on the purpose and procedure of the experiment, and signed a consent form. They were then compensated with a fee for their participation.

### 5.2. Stimuli

The emotional photos produced in Experiment 1 were used as stimuli for self-awareness evaluation. The avatars produced in Experiment 2 were used as stimuli to evaluate the awareness of others. To evaluate the ability to distinguish expressed emotions, four avatars with the same emotional intensity for each domain produced in Experiment 2 were randomly placed side-by-side. The stimuli were presented four times for sufficient comparison. 

### 5.3. Experimental Protocol

**Figure 7:**
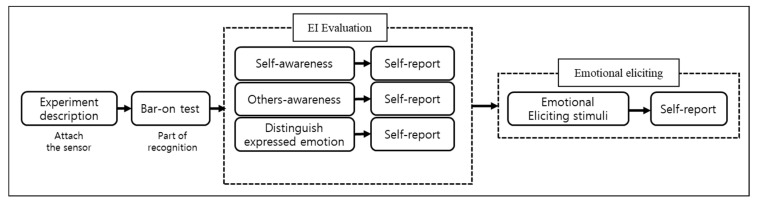
The overall experimental protocol of Experiment 4.

Figure 7 outlines the overall experimental protocol. To confirm the EI normality of the recruited participants, their self-recognition ability was evaluated using the EQ-i of the Bar-On model. The sum of the scores of three items (self-awareness, others-awareness, and distinguishing the expressed emotion) was used to evaluate the EI. Participants then viewed the emotion-eliciting stimuli.

The process for self-awareness evaluation was the same as that in Experiment 1. The process for others-awareness and distinguishing expressed emotion evaluations was the same as that in Experiment 2. However, in the questionnaire with regard to the evaluation to distinguish the expressed emotions, four randomly exposed avatars were selected among the four emotional domains (HAHV, HALV, LALV, LAHV) and neutral. To elicit emotional stimuli, four videos selected for each emotional domain were randomly displayed and a questionnaire was filled out. The questionnaire used was the same as that used in Experiment 1. All experimental procedures included the collection of ECG data. The score is 1200 points in total, with 10 points for each evaluation (3 items) * emotional domain (4 items) * the number of stimuli (10 items). Scores were given only when all problems in the evaluation items matched the target emotion. 

### 5.4. Results

#### 5.4.1. EI Normality of the Participants

**Figure 8:**
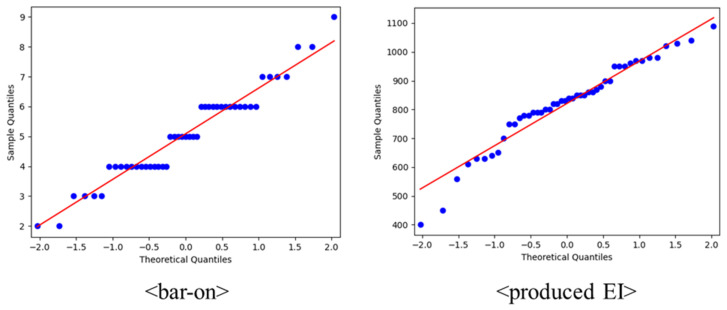
Normal distribution of participants by QQ plot.

To confirm the representativeness of the population’s EI, the normality of the existing EI evaluation (EQ-i) and the produced EI evaluation were verified. Figure 8 outlines the normal distribution of the two evaluations. The EQ-i showed *p* = 0.055, and the produced EI evaluation was *p* = 0.065, indicating normality in both evaluations. A *t*-test was used to compare HRV (and dynamics) in the EI group. 

#### 5.4.2. EI Classification Criteria

**Figure 9:**
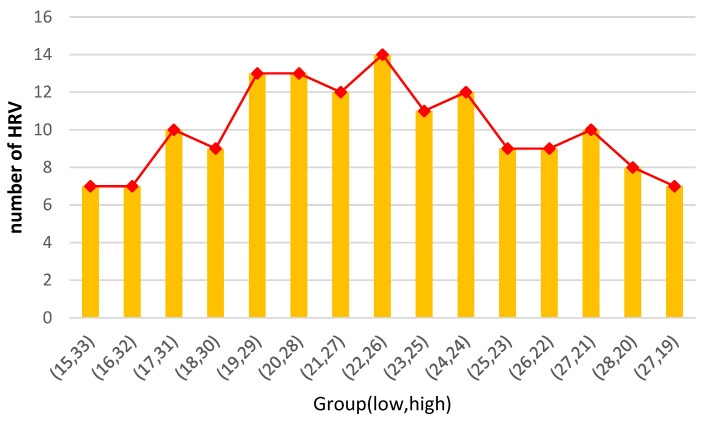
Change in the number of valid HRVs as a function of group composition between low and high groups.

Although the relative level of EI of the participants can be identified using the evaluation score, it is necessary to establish a robust criterion for classifying EI. In this study, the number of EI groups (low and high) was adjusted based on the evaluation score. The statistical effectiveness of HRV was analyzed using a *t*-test to establish the optimal classification criterion. We validated the number of occurrences of HRV indices and identified the distribution point of participant numbers between the low and high HRV groups (Figure 9). Based on the significant *p*-values of HF, lnHF, and RSA, which reflect emotional regulation, we confirmed the distribution point of the number of participants between the low and high HRV groups (Figure 9). Specifically, the highest number of significant HRV indices was 14 (Table 7), dividing 22 (low group) and 26 (high group) participants, which was 820 points (out of 1200) in terms of total scores. Overall, the number of valid HRV variables increased gradually, peaked, and then decreased. 

**Figure 10:**
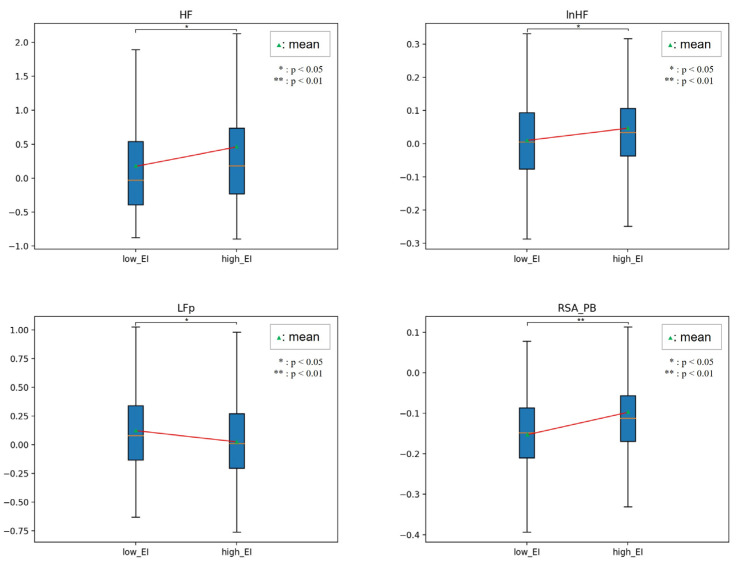
Comparison between low and high EI in HRV.

**Figure 11:**
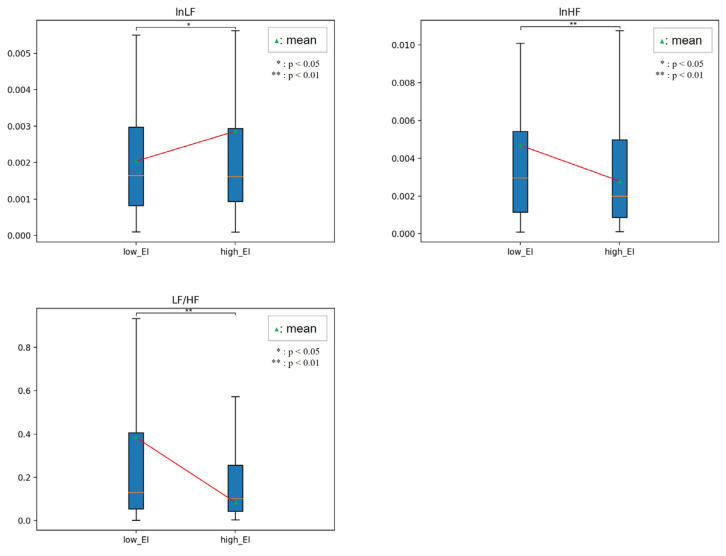
Comparison between low and high EI in variability.

**Figure 12:**
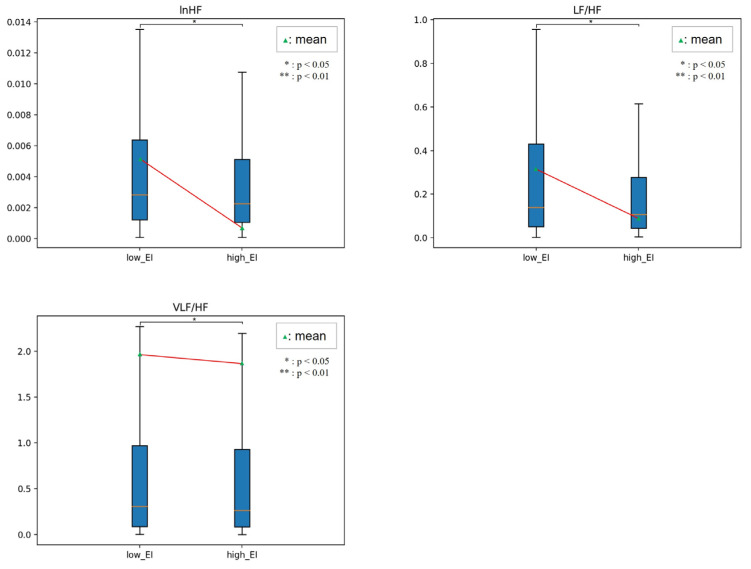
Comparison between low and high EI in instability.

**Figure 13:**
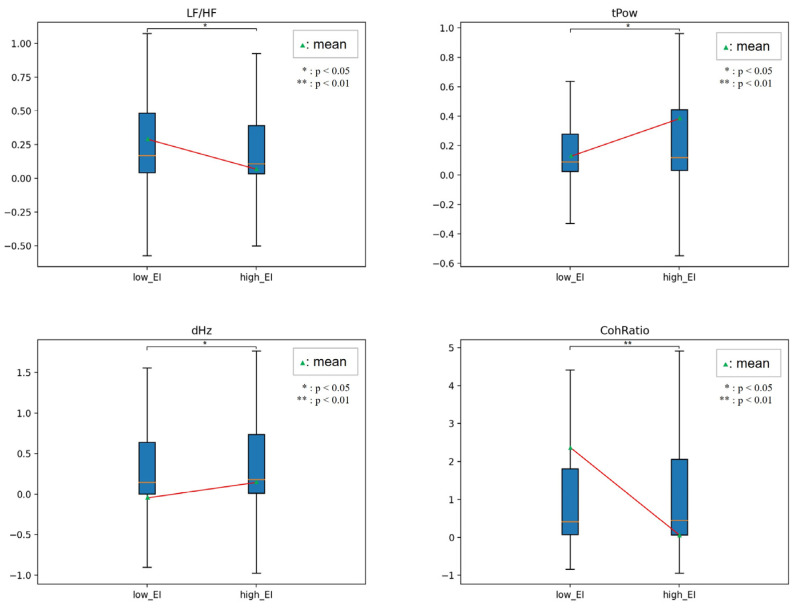
Comparison between low and high EI in inertia.

Figure 10, Figure 11, Figure 12 and Figure 13 show the HRV variables at the dividing point. The most effective HRV variable was found in the 22 low and 26 high groups; a total of 14 HRV variables were effective. 

Table 8 presents the analysis results for the two EI groups. In related EI studies, humans in the high EI group reported that HF, LF/HF, and RSA, reflecting vagal tone, were higher than those in the low EI group [32,33]. In our study, emotional regulation ability was not limited to stress or mental or neurological lesions. Instead, we used emotion-eliciting stimuli based on a two-dimensional emotional model. 

In this study, the high EI group had higher lnLF variability, lower HF variability, and lower lnHF of instability than those in the lower EI group. The results indicated that participants adapted rapidly to the external environment and implied that their ability to cope was high. The LF/HF of variability, LF/HF, VLF/HF instability, LF/HF, and CohRatio of inertia were all lower. These parameters show the magnitude of the ANS balance, which consistently shows that the high EI group maintains ANS homeostasis better than the low EI group does.

In addition, the total power and dominant frequency of the inertia were higher. These parameters indicate the degree of ANS activation, which means that the high EI group had a higher degree of instantaneous activation than the low EI group.

## 6. Discussion and Conclusions

Our study makes three primary contributions. Firstly, we assessed emotional intelligence (EI) levels using physiological measures such as heart rate variability (HRV) and dynamics. Secondly, we evaluated participants’ EI by assessing their recognition of stimuli based on a dimensional model of arousal and valence. Lastly, we increased the validity of our evaluation method by incorporating facial intensity and enhancing the granularity of our stimuli. To the best of our knowledge, this is the first study to utilize HRV in the analysis of EI.

To achieve such contributions, we conducted four intertwined experiments. In the first experiment, based on the IAPS model, we selected photos with a high average accuracy of 92.5%. In the second experiment, we standardized facial expressions using a two-dimensional emotional model with avatars and defined facial expression stimuli by normalizing the facial expression intensity. In the third experiment, we obtained HRV and dynamic data reflecting emotion regulation by producing and selecting emotion-eliciting videos containing highly valid facial expressions based on emotional contagion. Lastly, we evaluated participants’ EI levels through self-reports using photos and avatars while collecting ECG data as they watched the emotional video. We identified critical HRV and dynamics, which were then utilized to classify EI through analysis

We addressed the negative response distortion effect of questionnaire-based EI tests [8,9,11] by subdividing stimuli to achieve a high-resolution assessment of participants’ emotional recognition capability. To improve the accuracy of emotional regulation, we normalized HRV and dynamics to account for individual physiological differences. We utilized HRV and dynamics, involuntary indicators to analyze and validate our criteria for EI assessment. To the best of our knowledge, this is the first study to analyze HRV and dynamics to investigate EI.

Previous research on emotion recognition has relied heavily on subjective evaluations, but individual differences in EI can compromise the validity of these datasets. By accurately classifying EI levels, researchers can obtain more precise and valid data on emotion recognition. However, our study has limitations in that we generalized EI levels across all emotional domains, which may differ and require further investigation.

## Figures and Tables

**Table 2:** Two experiments to produce stimuli (photo and avatar) for the latter experiments.

Experiment	Purpose	Index	Perception Level	Method
#1	Selecting photo Stimuli	Self-awareness	Recognize one’s emotions (Level 1) Express one’s emotions (Level 3)	Participant evaluation
#2	Selecting avatar Stimuli	Others-awareness	Recognize other’s emotions (Level 2) Express one’s emotions (Level 3)	
		Discriminate emotion	Express one’s emotions (Level 3) Distinguish emotions accurately (Level 4)	

**Table 3:** Main experiments to measure HRV and classify EI.

Experiment	Purpose	Method
#3	Measuring physiological response to evaluate EI	Extraction of HRV and dynamics when participants watched the stimuli from experiments #1 and #2
#4	Identifying criteria for classifying EI	Comparison of statistical differences in HRV from experiment #3

**Table 4:** Results of the recognition accuracy of emotional photos in each emotional domain.

Emotional Domain	Accuracy (%)
HAHV	92%
HALV	98%
LALV	87%
LAHV	93%

**Table 5:** Results of highest accuracy for each emotional domain and corresponding intensity.

Emotional Domain	Intensity	Accuracy (%)
HAHV	3	100%
HALV	6	96.7%
LALV	6	86.7%
LAHV	5	86.8%

**Table 6:** Heart rate variability variables.

Type	Parameter	Description
Time domain	BPM (average heart rate per minute)	
	SDNN (ms)	Standard deviation of NN interval
	RMSSD (ms)	Root mean square of successive RR interval
	pNN50 (%)	Percentage of successive RR intervals that differ by more than 50 msec
	RSA	
Frequency domain	VLF	Absolute power in the 0.0033 to 0.04 Hz band
	LF	Absolute power in the 0.004 to 0.15 Hz band
	HF	Absolute power in the 0.015 to 0.4 Hz band
	lnVLF	VLF taken as natural logarithm
	lnLF	LF taken as natural logarithm
	lnHF	HF taken as natural logarithm
	VLF power (VLFp)	Relative power of VLF
	LF power (LFp)	Relative power of LF
	HF power (HFp)	Relative power of HF
	VLF/HF	VLF divided by HF
	LF/HF	LF divided by HF
	Total power (tPow)	Absolute power in the 0.0033 to 0.4 Hz band
	Dominant power (dPow)	Power of the highest peak in the power spectrum
	Total Hz (tHz)	Absolute power in the 0.0033 to 0.4 Hz band
	Dominant Hz (dHz)	Frequency of the highest peak in the power spectrum
	Coherence ratio (CohRatio)	Peak power divided by the differencebetween total power and peak power

**Table 7:** The 14 significant markers to discriminate high and low EI.

		Time Domain	Frequency Domain			Homeostasis
			Basic	Balance of ANS	Highest of HZ, Power	
HRV		-	HF *, lnHF *	LFp *	-	RSA **
Dynamics	Variability	-	lnLF *, lnHF **	LF/HF **	-	-
	Instability	-	lnHF*	LF/HF *, VLF/HF *	-	-
	Inertia	-	-	LF/HF *	tPow *, dHz *	CohRatio **

* *p*-value < 0.05: 10 Indices, ** *p*-value < 0.01: 4 Indices.

**Table 8:** The statistical comparison of HRV and dynamics between the two EI levels.

Type	Parameter	Low EI		High EI		*p*-Values
		Mean	STD	Mean	STD	
HRV	HF	0.235	0.758	0.443	0.976	0.031
	lnHF	0.014	0.111	0.041	0.107	0.041
	LFp	0.123	0.315	0.050	0.281	0.030
	RSA	−0.149	0.080	−0.112	0.086	0.002
Variability (Dynamics)	lnLF	0.0021	0.0003	0.0028	0.0022	0.026
	lnHF	0.0045	0.0041	0.0027	0.0024	0.009
	LF/HF	0.3890	0.4670	0.0776	0.082	0.001
Instability (Dynamics)	lnHF	0.0051	0.0035	0.0033	0.0027	0.044
	LF/HF	0.3992	0.4341	0.0938	0.0618	0.012
	VLF/HF	1.911	5.703	1.846	7.243	0.033
Inertia (Dynamics)	LF/HF	0.2783	0.3668	0.0610	0.4420	0.044
	Total Power	0.1444	0.3284	0.3865	0.4028	0.019
	Dominant Hz	−0.0672	0.5574	0.1788	0.4719	0.038
	Coherence Ratio	0.2329	0.3694	0.0027	0.3232	0.008

## Data Availability

Not applicable.
